# Functional Analysis of Water Stress-Responsive Soybean GmNAC003 and GmNAC004 Transcription Factors in Lateral Root Development in *Arabidopsis*

**DOI:** 10.1371/journal.pone.0084886

**Published:** 2014-01-23

**Authors:** Truyen N. Quach, Lam-Son Phan Tran, Babu Valliyodan, Hanh TM. Nguyen, Rajesh Kumar, Anjanasree K. Neelakandan, Satish Kumar Guttikonda, Robert E. Sharp, Henry T. Nguyen

**Affiliations:** Division of Plant Sciences, National Center for Soybean Biotechnology and Interdisciplinary Plant Group, University of Missouri, Columbia, Missouri, United States of America; National Taiwan University, Taiwan

## Abstract

In *Arabidopsis*, NAC (NAM, ATAF and CUC) transcription factors have been found to promote lateral root number through the auxin signaling pathway. In the present study, the role of water stress–inducible soybean *GmNAC003* and *GmNAC004* genes in the enhancement of lateral root development under water deficit conditions was investigated. Both genes were highly expressed in roots, leaves and flowers of soybean and were strongly induced by water stress and moderately induced by a treatment with abscisic acid (ABA). They showed a slight response to treatment with 2,4-dichlorophenoxyacetic acid (2,4-D). The transgenic *Arabidopsis* plants overexpressing *GmNAC004* showed an increase in lateral root number and length under non-stress conditions and maintained higher lateral root number and length under mild water stress conditions compared to the wild-type (WT), while the transgenic plants overexpressing *GmNAC003* did not show any response. However, LR development of *GmNAC004* transgenic *Arabidopsis* plants was not enhanced in the water-stressed compared to the well-watered treatment. In the treatment with ABA, LR density of the *GmNAC004* transgenic *Arabidopsis* was less suppressed than that of the WT, suggesting that *GmNAC004* counteracts ABA-induced inhibition of lateral root development. In the treatment with 2,4-D, lateral root density was enhanced in both *GmNAC004* transgenic *Arabidopsis* and WT plants but the promotion was higher in the transgenic plants. Conversely, in the treatment with naphthylphthalamic acid (NPA), lateral root density was inhibited and there was no difference in the phenotype of the *GmNAC004* transgenic *Arabidopsis* and WT plants, indicating that auxin is required for the action of *GmNAC004*. Transcript analysis for a number of known auxin and ABA related genes showed that *GmNAC004*'s role may suppress ABA signaling but promote auxin signaling to increase lateral root development in the *Arabidopsis* heterologous system.

## Introduction

Soybean root architecture is defined by tap root length and lateral root (LR) length, density and distribution. The tap root is produced during embryogenesis, while LRs are produced throughout the life of the plant. Optimum root architecture is the result of coordinated interaction between the genetic limit and environmental factors [1]. Environmental conditions include the soil water content and nutrient composition, soil physical properties such as particle size, compactness and porosity, soil temperature, and the living organisms around the plant. Regulation of root growth and development in response to environmental stresses to optimize water and nutrient extraction has been observed in various species including soybean [2]–[4], barley [5], *Arabidopsis* [6] and common bean [7], [8].

In soybean, early establishment of the root system is an important trait in the selection of genotypes for improvement of soybean production in drought-prone areas [9], [10]. Under mild water deficit conditions, root growth rate is relatively less inhibited or even stimulated, while shoot growth is significantly inhibited [2], [4], [11]. More extensive root growth and development occurs in deeper soil layers under conditions of soil drying [3], [4], [12], [13]. Increased LR development under mild water deficit conditions was reported in a majority of soybean genotypes from a collection of eleven varieties [4]. Primary root lengths of these lines remained unchanged while shoot growth was reduced. These data suggest that soybean plants can optimize resources to prioritize LR development to adapt to water stress.

LR development is regulated by various plant hormones and their interactions [14]–[16]. The phytohormone auxin, synthesized in young parts of the shoot and roots, is the primary hormone acting in the regulatory process [17]. The auxin signal is required at early events of pericycle cell selection to develop LR primordia [18], [19]. The accumulation of auxin induces expression of various genes in the auxin signaling pathways, and results in activating cell cycle proteins to direct cell division in the initiation step [20]. In the emergence step, auxin triggers cell wall remodeling enzymes to facilitate cell separation and cell shape modification [21]. Other plant hormones, including ethylene and abscisic acid (ABA), also play roles in the regulation of root growth in response to environmental changes. Ethylene has been shown to induce adventitious root development in response to low-oxygen stress in maize [22]. Recently, Ivanchenko *et al.* [23] reported that LR number in *Arabidopsis* was promoted by low concentrations of ethylene, but inhibited by higher concentrations of this hormone. ABA is accumulated under water stress and has been shown to help maintain primary root growth in maize by acting at least in part through the inhibition of excess ethylene production [24], [25]. In the regulation of LR development, however, ABA appears to inhibit various processes from initiation to emergence [26]. Exogenous ABA treatments suppress LR numbers in wild-type (WT) *Arabidopsis* plants [27], while ABA deficiency enhances LR growth in the ABA-deficient *aba2-1* and *aba3-1 Arabidopsis* mutants under both control and mannitol-induced osmotic stress conditions [28]. ABA acts antagonistically to auxin in the regulation of LR development at several stages including LR initiation and post-initiation [26].

The NAC (**N**AM-no apical meristem, **A**TAF- *Arabidopsis* transcription activation factor, and **C**UC-cup-shaped cotyledon) transcription factor (TF) family is a large plant-specific TF family. NAC TFs are involved in diverse processes including development, defense and abiotic stress responses [29]–[33]. Overexpression of several dehydration-inducible *NAC* genes resulted in improved drought tolerance in rice [34], [35] and *Arabidopsis* [36]. A number of NAC TFs can also act as positive regulators of LR development through regulation of auxin signaling pathways [37]–[39]. *Arabidopsis* NAC1 acts downstream of an auxin receptor, TIR1, to regulate several proteins in the auxin signaling pathway. Overexpression of the *NAC1* gene increased LR number and was able to restore the phenotype of the *tir1* mutant. Consistently, down-regulation of *NAC1* reduced LR development in transgenic *Arabidopsis* overexpressing the *TIR1* gene [39]. Another *Arabidopsis* NAC TF encoding gene, *AtNAC2*, which is induced by dehydration, ABA and salt stresses, was shown to increase LR number in transgenic plants [38]. Recently, soybean *GmNAC20* was reported to stimulate LR number in transgenic *Arabidopsis* and its action also appears to be involved in genes related to auxin signaling [37].

Soybean contains a large family of NAC TFs, with more than 150 members [40]–[45], among which at least 38 *GmNAC* genes are dehydration-inducible [46]. In our previous report, we characterized 31 *GmNAC* genes and identified nine dehydration-inducible *GmNAC* genes [47]. Among these nine genes, only *GmNAC003* and *GmNAC004* showed differential expression patterns in response to ABA treatment, indicating that these two genes may be important regulators for soybean adaptation to water stress in an ABA-dependent manner. *GmNAC003* and *GmNAC004* were also shown to specifically express in roots, suggesting that they might play a role in the regulation of growth and development of the root system in response to water deficit conditions. These two TFs were localized in the nucleus and showed the ability to activate transcription in a yeast assay [47], [48]. With the hypothesis that water stress-inducible *GmNAC* genes might have a role in the enhanced development of LRs in soybean under mild water stress, the objective of the present study was to investigate the functions of *GmNAC003* and *GmNAC004* in LR development. The heterologous *Arabidopsis* system was selected for the study because of ease of genetic manipulation and available downstream hormone signaling information. In fact, the *Arabidopsis* model has been used successfully for functional characterization of *Arabidopsis* NAC1 and AtNAC2 and soybean GmNAC20 in stimulating lateral root development [37]–[39]. In the present study, we found that *GmNAC004* and *GmNAC003* had an increased expression level under auxin treatment and promoted LR number and total LR length in transgenic *Arabidopsis* plants under well-watered conditions. Transgenic *Arabidopsis* plants overexpressing *GmNAC004* maintained a higher LR number and greater total LR length compared to the WT under mild water deficit, but there was no promotion of LR development under water deficit conditions when compared to the well-watered control. GmNAC004 appears to regulate LR development in *Arabidopsis* under normal growth conditions via suppression of ABA signaling and promotion of auxin signaling.

## Materials and Methods

### Stress and hormone treatments of soybean and tissue collection

#### Dehydration, cold, NaCl, ABA and 2,4-D treatments

Soybean cv. Williams 82 (W82) was grown in a greenhouse (60% relative humidity, 28/20°C day/night temperature, 14/10 h photoperiod, and ∼800 µmol m^−2^ s^−1^ photosynthetic photon flux) with a density of four to five plants per 1.5-gallon pot, which contained a mixture of sand and turface in a 1∶1 ratio. The plants were watered every two days. When the plants reached the V1 growth stage (first fully open trifoliate leaf), they were carefully harvested and then transferred to stress conditions. For dehydration treatment, harvested plants were placed on paper towels in a growth chamber with the following conditions: 75% relative humidity, 22/18°C day/night temperatures and ∼200 µmol m^−2^ s^−1^ photosynthetic photon flux. For cold treatment, the plants were placed with their roots submerged in cold water (4°C) and maintained in a cold room at the same temperature. For hormonal and NaCl treatments, the roots were submerged in solutions containing either 100 µM ABA, 10 µM 2,4-D, or 250 mM NaCl, as previously described [39], [41]. A water treatment was used as the control because all chemicals and cold stresses were imposed in aqueous solutions. Three individual plants were sampled after 1, 2, 5, 10, and 24 h of the various treatments.

#### Water stress treatments

The water stress treatment was imposed when the plants reached the V1 growth stage (day 14) by withholding water until the desired stem water potentials of −0.5 MPa (∼ day 18), −1.0 MPa (∼ day 22) and −1.5 MPa (∼ day 34) were obtained (Table S1). At each harvest, corresponding well-watered plants that had a stem water potential of ∼ −0.25 MPa were harvested and used as temporal controls. To measure pre-dawn (∼4 AM) stem water potentials, the stems were excised at the middle of the hypocotyl and water potential was immediately measured using a pressure chamber (PMS Instrument Co., Albany, CA, USA). Pre-dawn tissue collection was selected for water potential measurements because transpiration is at the minimum and the stem water potential is closest to soil water potential at this time.

### RNA isolation, cDNA synthesis and quantitative real-time PCR (qRT-PCR)

The *GmNAC003* (DQ028771) and *GmNAC004* (DQ028772) gene specific primers were designed using Primer 3 software [49] and are listed in Table S2. The primers were verified for binding specificity by blasting each primer sequence against the soybean genome sequence information at Phytozome (http://www.phytozome.net). For transcript analysis of *GmNAC003* and *GmNAC004* in response to drought, abiotic stresses and hormonal treatments, nine soybean internal controls (Table S2) were included for the qRT-PCR reactions and the most stable genes were selected to calculate the normalization factors using GeNorm [50]. RNA isolation, cDNA synthesis and qRT-PCR were performed as previously described [47], [51]. The qRT-PCR data were analyzed with the SDS 2.3 software package and a common signal threshold was set to 0.1. PCR amplification efficiency was obtained using the assumption-free PCR efficiency calculated by LinRegPCR [52]. Relative RNA abundance of each gene was calculated as GOI/N, where GOI is the expression quantity of the gene of interest and normalization factor. N was calculated by GeNorm as the geometric mean of the selected internal reference genes. In the situation where multiple controls were present, the treatment with the lowest expression was transformed to a value of 1 for convenient comparisons. For quantification of auxin and ABA related genes in *Arabidopsis*, ubiquitin was used as a reference gene (Table S2) and transcript profiling was performed for root samples collected from plants grown on ¼-strength MS nutrient agar plates.

### Construction of transgenic *Arabidopsis* plants

The open reading frames of soybean *GmNAC003* and *GmNAC004* genes were cloned by Tran *et al.* [47]. The two genes were subsequently inserted into a pGreen plasmid that had been pre-inserted with the 35S promoter, to produce pGreen-P35S-GmNAC003 and pGreen-P35S-GmNAC004. These binary vectors containing the soybean *GmNAC* genes were verified by sequencing and transformed into *Agrobacterium tumefaciens* strain C58, which had been transformed with the helper vector pSoup. All binary plasmid constructs were transformed into *Arabidopsis* using the floral dip method [53]. Transgenic plants at T1, T2, and T3 generations were screened in ¼-strength MS medium containing either 25 mg/L hygromycin. Transgenic plants were verified for T-DNA insertion by PCR and the expression of *GmNAC003* and *GmNAC004* was determined by qRT-PCR. Two events having single insertion copy and comparable plant growth to the WT were selected for phenotypic assays of root development. Transgene expression in the transgenic *Arabidopsis* plants was quantified from 2-week old seedlings grown on ¼-strength MS nutrient agar plates. The *Arabidopsis* ubiquitin gene (AT3G62250) was used as the reference gene for the qRT-PCR with the primers listed in Table S2.

### Germination assay

Sterilized *Arabidopsis* seeds were sown at a density of 50–70 seeds on basal nutrient plates, either with 1 µM ABA (ABA treatment) or without ABA (control treatment). The plates were incubated in dark at 4°C for 4 days for stratification and were then placed in a growth chamber with 70% relative humidity, 22°C constant temperature, 16/8 hour day/night, and ∼100 µmol m^−2^ s^−1^ photosynthetic photon flux. Seeds with visible roots were counted after 5 days under normal growth conditions [54]. The germination rate was calculated from the total number of seeds sown, and was normalized to the germination rate of the control plates.

### *Arabidopsis* root growth assays under low water potential and hormonal treatments

*GmNAC003* and *GmNAC004* transgenic and WT *Arabidopsis* plants were subjected to root-growth assays in response to water stress and hormonal treatments on agar plates. The agar plates contained basal nutrient medium plus 1.2% Difco bacto agar. The basal nutrient medium comprised ¼-strength MS and 0.5 g/L monohydrate 2-N-morpholino ethanesufonic acid (MES), and was adjusted to pH 5.72. No sucrose was added to avoid any interference of sugar on growth and molecular signaling [55].

#### Water stress treatment

Polyethylene glycol (PEG MW8000, P5413, Sigma Inc., St Louis, MO, USA) was used to lower the water potential of the medium. The diffusion of PEG into the nutrient agar plates was conducted as described by van der Weele *et al.* [49] using 245×245 mm bioassay plates (431272, Corning, NY, USA). One hundred sixty ml of basal nutrient medium solution containing 0%, 10%, 15% or 20% PEG were poured onto plates containing an equal amount of solidified nutrient agar media to create a range of mild water stress conditions. The plates were gently shaken for 16 hours to facilitate PEG diffusion and equilibrate the water potentials. Excess PEG solution was then completely evacuated from plates using a vacuum pump.

*Arabidopsis* seeds were surface-sterilized with 70% ethanol for 2 minutes followed by 10% bleach for 2 minutes and then rinsed four times with distilled water. After stratification for four days at 4°C in darkness, the seeds were transferred to a nursery plate and treated with red light for 1 hour and then kept in the dark for 24 hours at 22°C to promote germination. The plates were then placed in a growth chamber in a vertical position to allow downward root growth for three days. At day 4 (4 days after sowing), seedlings with a primary root length between 9 and 11 mm were transferred to bioassay plates with designated PEG concentrations as described above. Each bioassay plate contained twenty-five seedlings from five genotypes (two transgenic GmNAC003, two transgenic GmNAC004, and the WT lines). The plates were sealed by two layers of micropore tape (Micropore, 3M Company, St Paul, MN) and placed vertically in a growth chamber (Conviron model A1000TC) at a constant relative humidity of ∼70%, 22/18°C day/night temperatures, 16/8 hour day-length, and ∼200 µmol m^−2^ s^−1^ photosynthetic photon flux. At day 12, the plates were measured for LR number, total root length and primary root length. LR number was counted visually using a magnifying glass. Total root length was measured using the WinRHIZO program (Regent Instruments Inc., QC, Canada). Primary root length was measured with a ruler from the root/shoot junction to the root tip. Total LR length was calculated by subtracting the primary root length from the total root length. The experiment was repeated three times with six replicated plates to check for consistent results. At the end of the experiment, water potentials of the agar plates were measured using isopiestic thermocouple psychrometry [56].

#### Hormone treatments

Seedlings of GmNAC004 transgenic plants were grown as described above for the PEG treatments. At day 4 (4 days after sowing), they were transferred to bioassay agar plates containing the indicated hormone(s) with a density of 25 seedlings per plate. Each hormone treatment contained the basal nutrient medium (described above) with added single or combination of hormones to final concentrations of 5 µM ABA, 20 nM 2,4-D, and 2 µM NPA. The experiment was repeated two times with six replicated plates. LR number and length were measured at day 12 as described for the water stress treatments. NPA and 2,4-D have been reported to inhibit primary root elongation mainly by suppression of cell production [57]. Therefore, we used LR density rather than total LR number to compare LR development among treatments.

## Results

### *GmNAC003* and *GmNAC004* are induced by water stress

To investigate the response of *GmNAC003* and *GmNAC004* to water deficit in various soybean organs, a greenhouse experiment was conducted in which water was withheld to allow progressive water stress development to mimic slow soil-drying under drought conditions in the field. Tissues were collected at three water stress levels of ∼−0.5, −1.0 and −1.5 MPa stem water potentials, corresponding to 4, 8 and 20 days after water deficit treatment started (Table S1). These stress levels represent mild, intermediate and severe stress, respectively. The control soybean plants grew at a rate of about one new leaf per 3 days, and the developmental stages of the controls were different from the stressed plants, which almost ceased their growth as the stress intensified and had only 2 or 3 trifoliolate leaves by the end of the experiment. To avoid this difference in development, two controls were required: a developmental control having the same growth stage as the stressed treatments and temporal controls with the same durations of growth as the stressed treatments. The control V2 stage was considered a developmental control because the stressed plants did not produce any more leaves. For the temporal comparison, V2 was the control for −0.5 MPa, and similarly, V3 for −1.0 MPa and V6 for −1.5 MPa (Table S1). We define a water stress-responsive gene as a gene whose expression changed under water stress relative to both the developmental and temporal controls.

**Figure 1:**
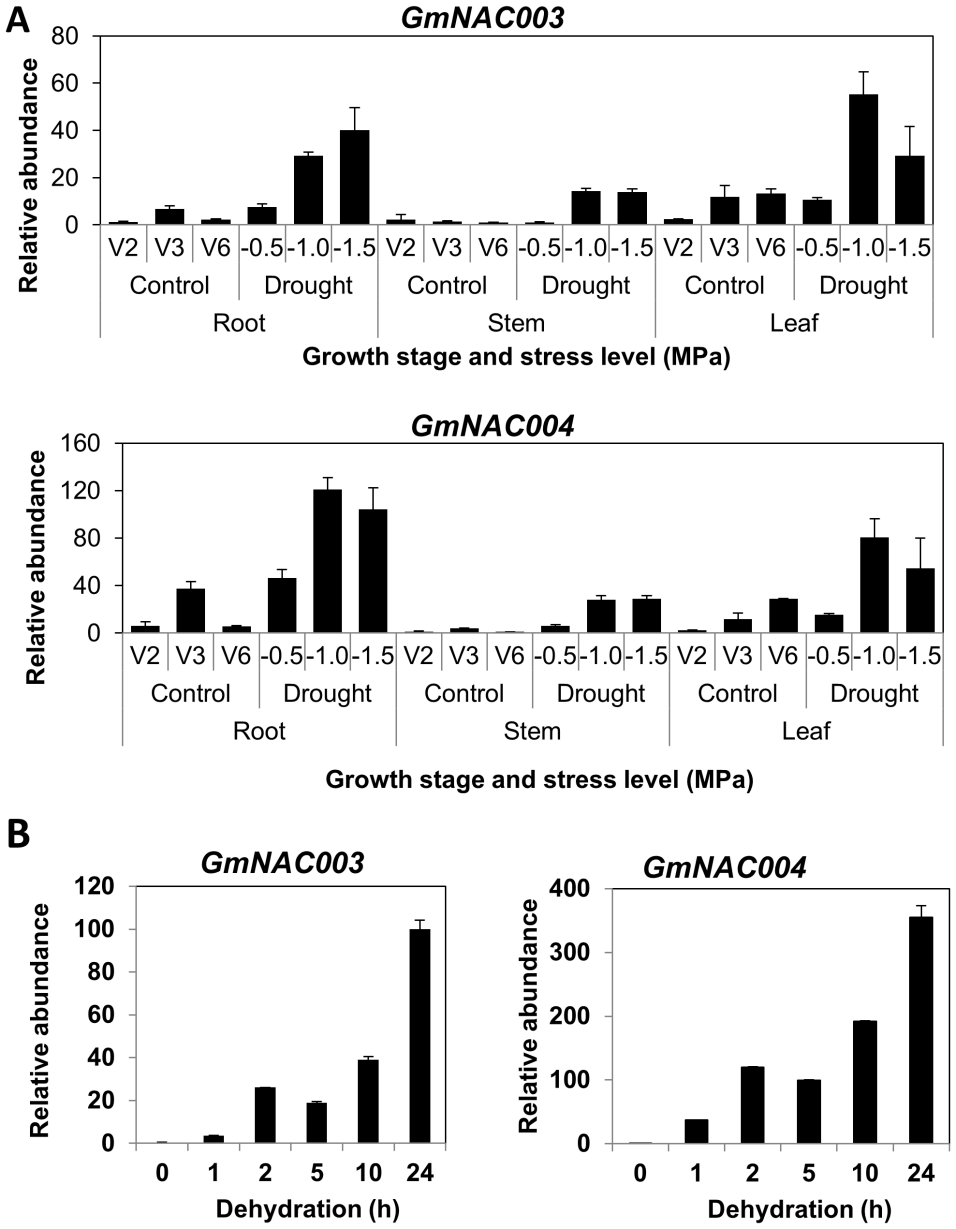
Relative transcript abundance of *GmNAC003* and *GmNAC004* in response to water stress. Transcript abundance was quantified using qRT-PCR and the data were normalized to the four best internal control genes (*CYP2, IDE, UNK1* and *UNK2*) based on the M stability of GeNorm analysis. Mean relative expression levels were transformed to a value of 1 for the sample having lowest expression. Error bars are standard errors of the means from three replications. (A) Response to water deficit stress. Plants at the 2-leaf (V2) growth stage were allowed to grow without supply of additional water until desired stem water potentials were achieved. V2, V3 and V6 are the growth stages of the control plants corresponding to the time when water stressed-plants were sampled. (B) Response to dehydration stress. V2 growth stage plants were harvested and allowed to dehydrate in a growth chamber for the designated times.

Figure 1A shows that the expression of *GmNAC003* and *GmNAC004* was strongly induced by water stress in all the tissues examined and that the inducibility of *GmNAC004* was stronger than that of *GmNAC003*. In root and leaf tissues, both genes responded significantly even at the mild water stress level (−0.5 MPa), while in the stem they were induced to a lesser extent and only at the intermediate and severe water stress levels.

To further examine the sensitivity of the response, we imposed dehydration stress by placing young soybean plants at the V1 growth stage on paper towels for 0, 1, 2, 5, 10 and 24 h. The severity of the stress was measured using a pressure chamber for stem water potentials and recorded as followed: −0.25 MPa for control, −0.83 MPa for 1 h, and −2.8 MPa for 5 h dehydration treatment. Water potentials of the plants that had been dehydrated for 10 h and 24 h were too low to be measured using the pressure chamber. Figure 1B shows that expression of *GmNAC003* and *GmNAC004* was induced as early as 1 h from the onset of dehydration by 4-fold and 37 fold, respectively. The inducibility of *GmNAC003* and *GmNAC004* transcription peaked at 24 h of dehydration. Consistent with the results of the slow soil-drying treatments shown in Figure 1A, *GmNAC004* showed a higher degree of inducibility than *GmNAC003*.

### Response of *GmNAC003* and *GmNAC004* to hormonal and stress treatments

**Figure 2:**
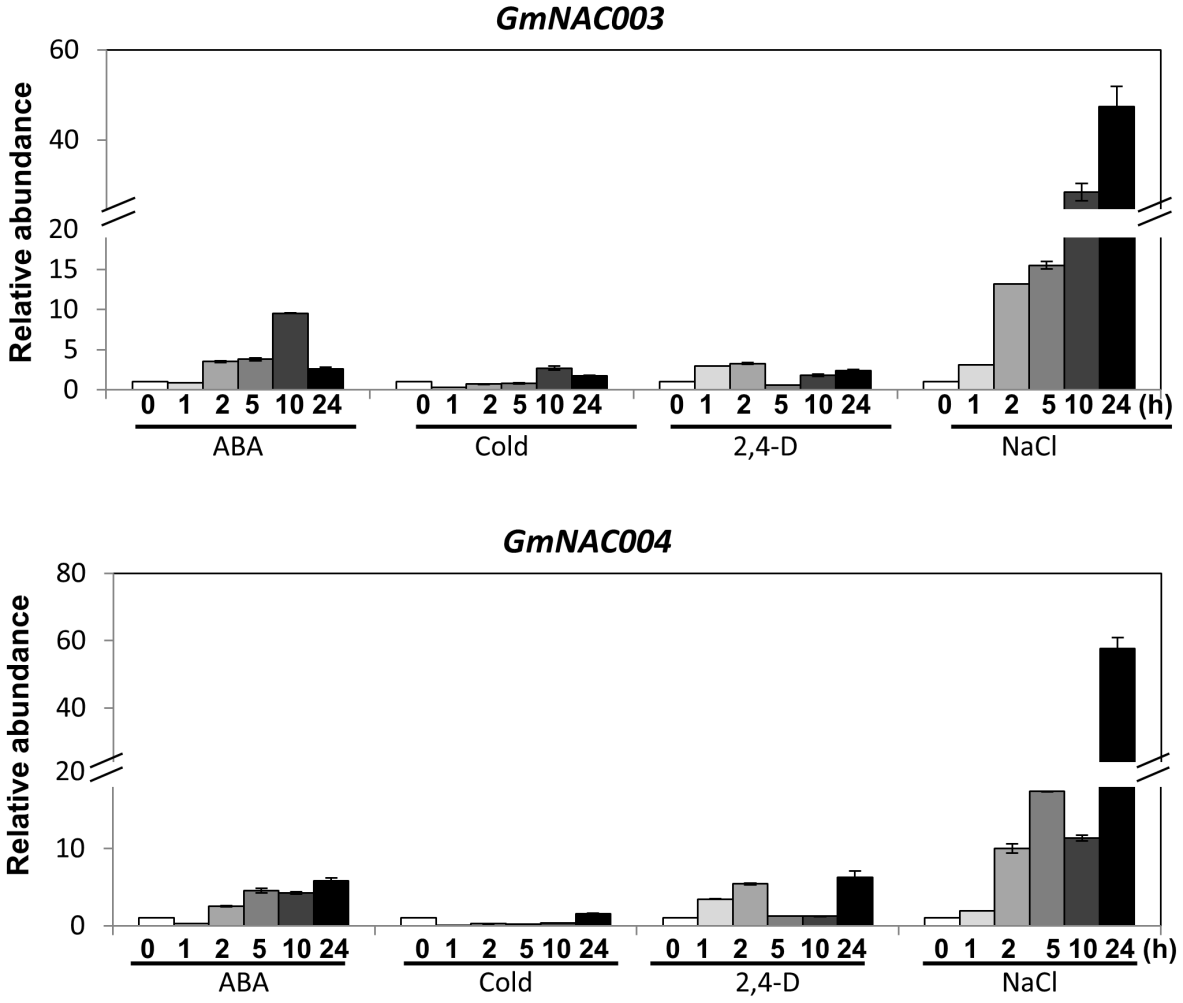
Expression of *GmNAC003* and *GmNAC004* in response to various stresses and hormonal treatments. V2 growth-stage soybean plants were harvested and transferred to stress conditions. All treatments of control (Water), 20 nM 2,4-D (2,4-D), 100 µM ABA (ABA), 4°C (Cold) and 250 mM NaCl (NaCl) were under hydroponic conditions at 28°C unless otherwise indicated. Transcript abundance was quantified by qRT-PCR and the data were normalized to the four most stable internal reference genes (*CYP2, IDE, UNK1* and *UNK2*) using GeNorm. Data were normalized using water treatments, and transformed to a value of 1 for the unstressed plant (0 h). The error bars are standard errors of the means (n = 3 plants).

Plant growth and development are regulated by various hormones and other environmental stresses, such as salinity and cold. In the abiotic signaling network, several *NAC* genes were reported to have ABA-dependent regulation of stress responses [34], [36], [58]. Although the expression of *GmNAC003* and *GmNAC004* under ABA, salinity and cold treatments was known in soybean cv. Maverick [47], we wanted to re-examine the expression of the *GmNAC003* and *GmNAC004* in W82, which was reported to have a greater LR number increase than cv. Maverick in response to mild water stress [4]. Figure 2 shows that both *GmNAC003* and *GmNAC004* genes showed higher expression under the NaCl treatment, with a similar trend observed in response to dehydration (Figure 1B). ABA treatment resulted in a moderate increase in gene expression of both genes. In contrast, cold stress had a slight increased expression of *GmNAC003* while suppressed expression of *GmNAC004* at early time points. The trend of responses of the *GmNAC003* and *GmNAC004* genes in W82 is consistent with our previous study which was conducted for Maverick, although the inducibility of *GmNAC003* and *GmNAC004* genes appeared to be higher in W82 than in Maverick in the salt stress treatment (Figure 2, [47]). The response to NaCl and dehydration treatments was detected as early as after 1 h of treatment. On the other hand, significant accumulation of *GmNAC003* and *GmNAC004* under ABA treatment was observed only after 2 h. Taken together, these data suggest that these *GmNAC* genes do not require ABA accumulation for their early responses to either NaCl or dehydration, and that they regulate responses to water deficit and salt stress in both an ABA-dependent and an ABA-independent manners.

NAC TFs have been shown to associate with auxin signaling in regulation of LR development [38], [39]. In our previous study, *GmNAC003* and *GmNAC004* had preferential expression in roots [47] suggesting that they may be involved in root development. To determine whether *GmNAC003* and *GmNAC004* act downstream of the auxin signaling pathway, we investigated their expression in response to 2,4-D treatment. Figure 2 shows that expression of the *GmNAC003* gene in response to 2,4-D was slightly increased, while there was a moderate increase in expression of the *GmNAC004* gene after 1 h of 2,4-D treatment. The response of *GmNAC004* was rapid but transient for the first few hours, and then the expression was regained after a longer time of treatment.

### *GmNAC003* and *GmNAC004* sequences are closely related to lateral root-stimulating *GmNAC20* protein

To understand sequence relationship among GmNAC003 and GmNAC004 and GmNAC20 [37] we reconstructed a phylogenetic tree of 152-soybean NAC transcription factor family (Figure S1) using recently updated data [46]. GmNAC003 and GmNAC004 have high sequence similarity (67% identical amino acid sequences for full length and 92% for the 170-amino acid conserved NAC domain). However, these two proteins are relatively different at carboxyl terminals which acted as an activating domain [47]. These two proteins are sub-grouped to the same branch with GmNAC20 and NAC domain sequence similarity between GmNAC004 and GmNAC20 is 66%. GmNAC003, GmNAC004 and GmNAC20, however, has low sequence similarity with *Arabidopsis* NAC1 and AtNAC2, which function to stimulate LR number in *Arabidopsis* [38], [39].

### *GmNAC004* stimulates LR numbers and total LR length in transgenic *Arabidopsis* at high water potentials

**Figure 3:**
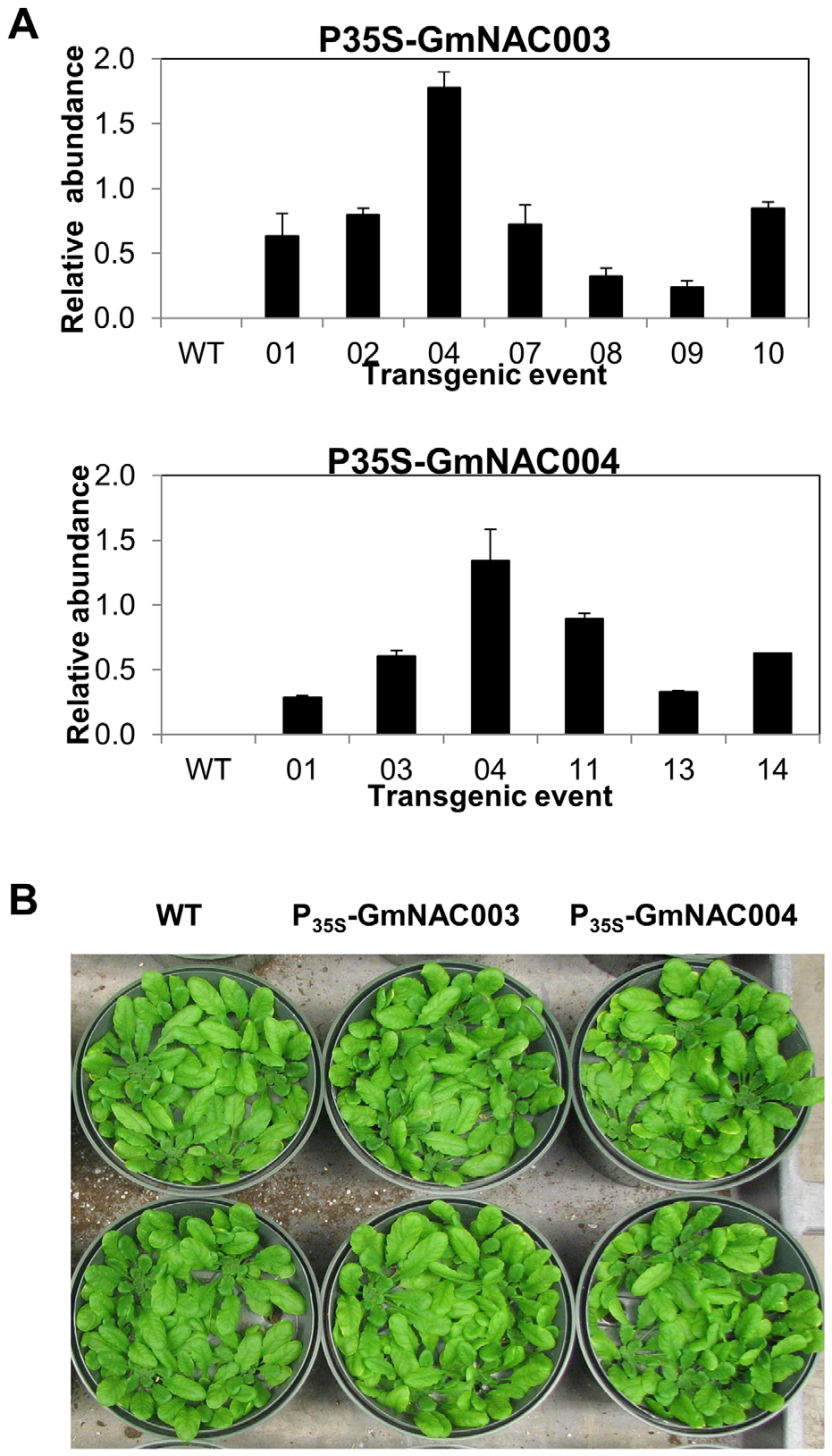
Transgenic *Arabidopsis* plants overexpressing soybean *GmNAC003* and *GmNAC004*. (A) Expression of the transgenes in 3-week-old transgenic plants quantified by qRT-PCR using *Arabidopsis* ubiquitin as the reference gene. The tissues were sampled from homozygous transgenic plants having a single insertion. Error bars are the standard errors of the means from three samples of ten plants. (B) Growth of the 4-week old and T4 generation *GmNAC003* (events N3.09 and N3.10) and *GmNAC004* transgenic (events N4.01 and N4.03) and WT plants.

**Figure 4:**
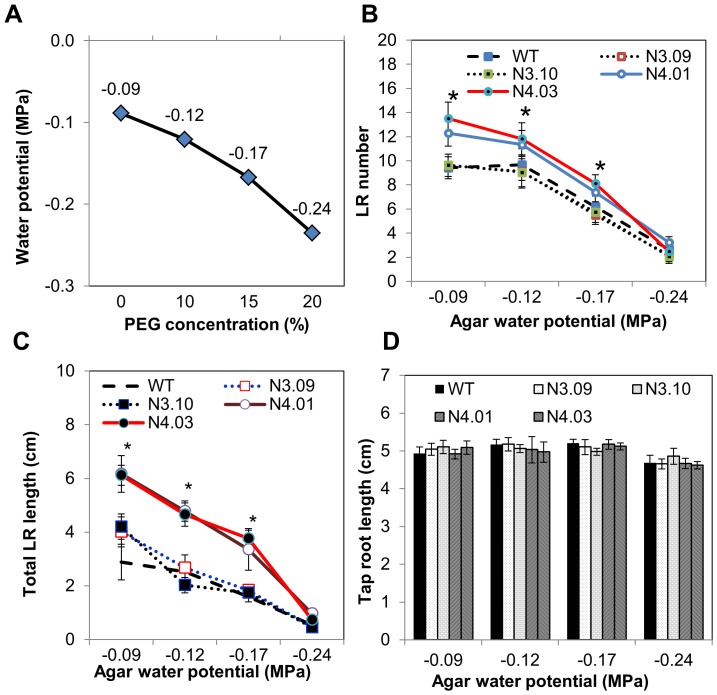
Lateral root numbers, lateral root length and primary root length of transgenic *Arabidopsis* plants overexpressing *GmNAC003* and *GmNAC004* in response to mild water stress. (A) Water potentials of PEG-diffused agar plates. Water potentials were measured at the end of the experiment, 8 days from the date of stress exposure. (B) Lateral root number, (C) total lateral root length, and (D) primary root length of transgenic plants. Transgenic *GmNAC003* (events N3.09 and N3.10), *GmNAC004* (events N4.01 and N4.03), and WT *Arabidopsis* lines were grown in nutrient agar plates diffused with different concentrations of PEG. The transgenic plants were at the T4 generation and homozygous for the transgenes. Data were recorded at 12 days after sowing (or 8 days of stress exposure). (*) denotes significant difference at 95% confidence level using Duncan's multiple range test from six replications. Error bars are standard errors of the means from 6 replications.

To explore the function of GmNAC003 and GmNAC004 in LR development we overexpressed the two genes in the *Arabidopsis* model. We used the *Arabidopsis* model for our work because of the ease of genetic manipulation and the previously successful characterizations of the *Arabidopsis* NAC1 and AtNAC2 TFs and the soybean GmNAC20 in the regulation of LR numbers [38], [39]. In addition, because *GmNAC004* was induced by water stress and by auxin treatment, we wanted to investigate whether this gene is involved in the enhancement of LR development under water stress. For this purpose, we developed transgenic *Arabidopsis* overexpressing *GmNAC003* and *GmNAC004* genes under control of the constitutive 35S promoter. Transgenic plants with single locus insertion copy were selected based on a 3∶1 segregation ratio at the T2 generation and used to develop T3 homozygous lines. Seven *GmNAC003* and six *GmNAC004* homozygous transgenic lines at the T3 generation having a single transgene insertion were selected for verification of transgenic expression and growth observation. Among the lines expressing the transgenes (Figure 3A), two homozygous lines at the T3 generation from each gene construct (lines 9 and 10 for *35S:GmNAC003* and lines 1 and 3 for *35S:GmNAC004*, Figure 4B) that showed shoot growth equivalent to the WT (Figure 3B) were selected for root growth assays. We generated several mild water deficit conditions within a water potential range from −0.09 to −0.24 MPa by diffusing PEG into the agar medium (Figure 4A). The water potentials of PEG-diffused agar plates were reproducible and constant throughout five days in growth chamber conditions, allowing accurate measurement of the stress level at the end of the experiments [59]. The range of water deficit in this study was comparable to the stress level in which He [4] reported promotion of LR number and length in soybean. This water deficit range, therefore, was considered suitable for the investigation of the roles of *GmNAC003* and *GmNAC004* genes in the regulation of LR growth and development under water stress.

Figure 4B showed that there was no promotion of LR number in *Arabidopsis* under mild water-stress conditions. Adding 10% PEG to the agar plates reduced the water potential from −0.09 to −0.12 MPa and caused a slight reduction in LR number. When the water potential was reduced to −0.17 MPa, suppression of LR number was significant in both WT and transgenic plants. At the lowest water potential of −0.24 MPa, LR number was greatly inhibited. This trend is consistent with findings reported previously for *Arabidopsis* [28], [59], which demonstrated that small changes in water potentials suppress LR formation, but is in contrast to soybean, which exhibited increased LR number and total length under mild water stress when grown on vermiculite medium [4].

**Figure 5:**
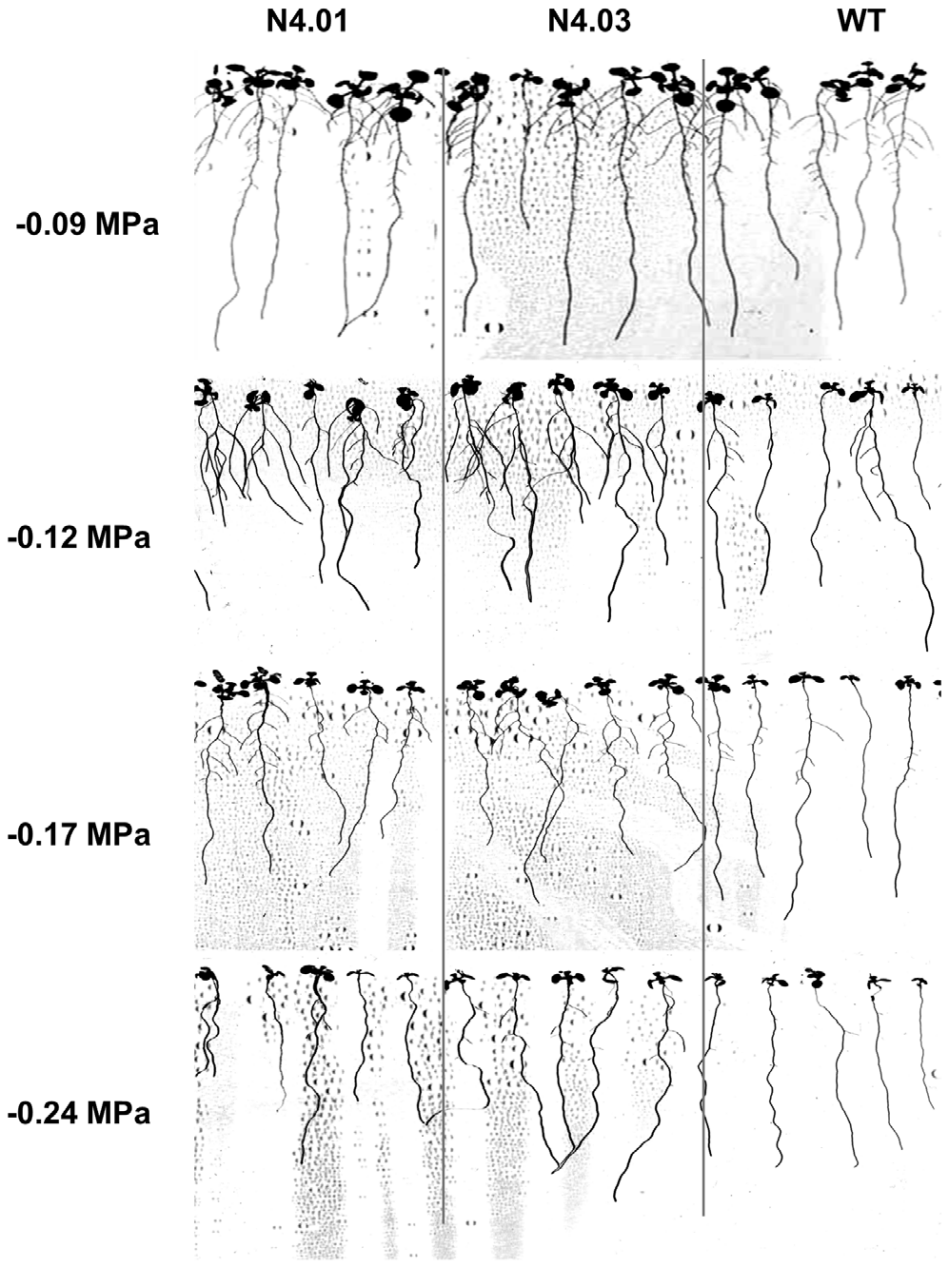
Representative root growth of transgenic *Arabidopsis* plants overexpressing *GmNAC004* in response to water deficit conditions. Two T4 homozygous transgenic *Arabidopsis* (N-4.01 and N-4.03) and WT plants were grown on nutrient agar plates diffused with different concentrations of PEG. The plants were 12 days old (8 days after stress exposure).

Constitutive overexpression of *GmNAC004* resulted in an increase in the number and total length of LRs in transgenic *Arabidopsis* plants compared to the WT (Figures 4B and 5), while that of *GmNAC003* had no effect on these traits (Figure 4B). Under well-watered conditions (−0.09 MPa water potential), transgenic *GmNAC004* plants exhibited an increase in LR number of 37% over the WT. Under the mild water deficit condition, a significant reduction in LR number was observed in both the transgenic and the WT plants; however, the *GmNAC004* transgenic plants still had 20–25% more LRs than the WT. Further lowering the water potential to −0.24 MPa strongly suppressed the LR number in both transgenic and WT plants. These data demonstrate that overexpression of *GmNAC004* increased LR number under non-stress conditions and maintained higher LR number under mild water stress conditions in comparison with the WT. Similar to LR number, total LR length was also increased in the *GmNAC004* transgenic plants compared to the WT under both well-watered (increased by 112%) and mild water stress (increased by 87–123%) conditions (Figure 4C). Although having similar expression profiles and a conserved DNA binding domain with those of *GmNAC004* (Figures 1&2 and [47]), overexpression of *GmNAC003* did not result in promotion of LR number or total LR length in transgenic *Arabidopsis* plants in comparison with the WT.

Within the range of mild water stress conditions from −0.12 to −0.24 MPa, primary root length was only slightly affected in both transgenic and WT *Arabidopsis* plants. Among the *Arabidopsis* lines, transgenic plants overexpressing *GmNAC003* and *GmNAC004* did not show significant changes in primary root length when compared to the WT at any tested water potentials (Figure 4D). Lowering the water potential to −0.24 MPa slightly decreased primary root length. This finding is consistent with the result reported by van der Weele *et al.* [59] where the authors found only a small change in primary root length in response to mild water stress.

### *GmNAC004* counteracts the ABA-induced inhibition of seed germination

**Figure 6:**
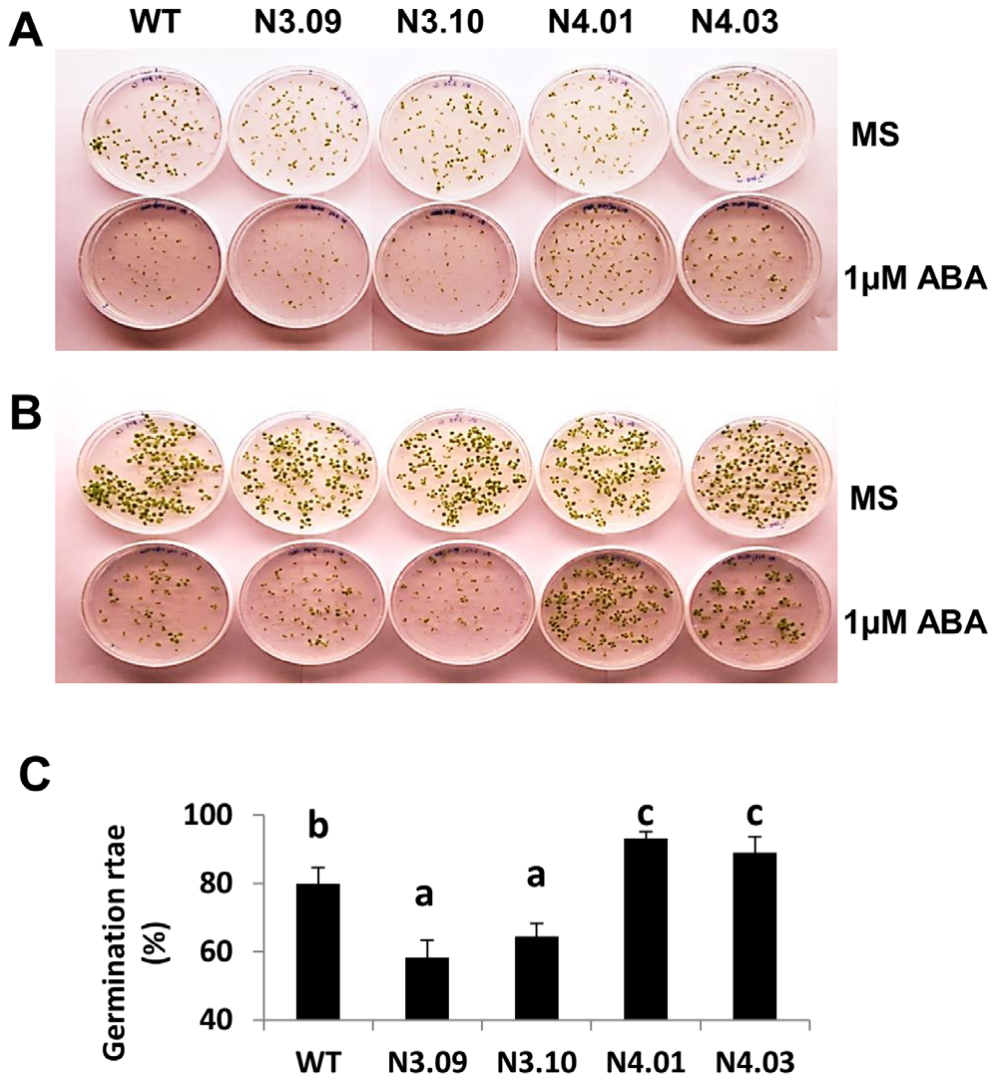
Germination of GmNAC003 and GmNAC004 transgenic *Arabidopsis* seeds in response to ABA treatment. Fifty to seventy seeds of the homozygous GmNAC003 and GmNAC004 transgenic and WT plants were sown on agar plates without ABA (MS) or with 1 µM ABA (ABA). (A & B) Plants were 7 and 14 days old, respectively. (C) Quantification of germination rates 5 days after sowing. Different letters denote significant differences at the 95% confidence level using Duncan's multiple range test from four replications.

Because the *GmNAC004* gene was induced by both ABA and water stress (Figures 1 and 2), GmNAC004 may regulate LR development via an ABA-signaling pathway. ABA is known to inhibit LR development [26] and suppress seed germination [60]. To investigate the involvement of GmNAC004 in the ABA-signaling network, we examined the response of seed germination of two transgenic events for each gene to a treatment of 1 µM ABA. Figure 6 showed that seed germination of the WT was inhibited by nearly 25% in the ABA treatment. The *GmNAC003* transgenic plants showed significantly higher inhibition, suggesting that GmNAC003 is a positive regulator of ABA signaling. By contrast, the *GmNAC004* transgenic plants displayed less inhibition in the presence of ABA. These data suggest that GmNAC004 counteracts ABA signaling, at least in seed germination.

### *GmNAC004* interacts with ABA and auxin signaling to regulate LR number

**Figure 7:**
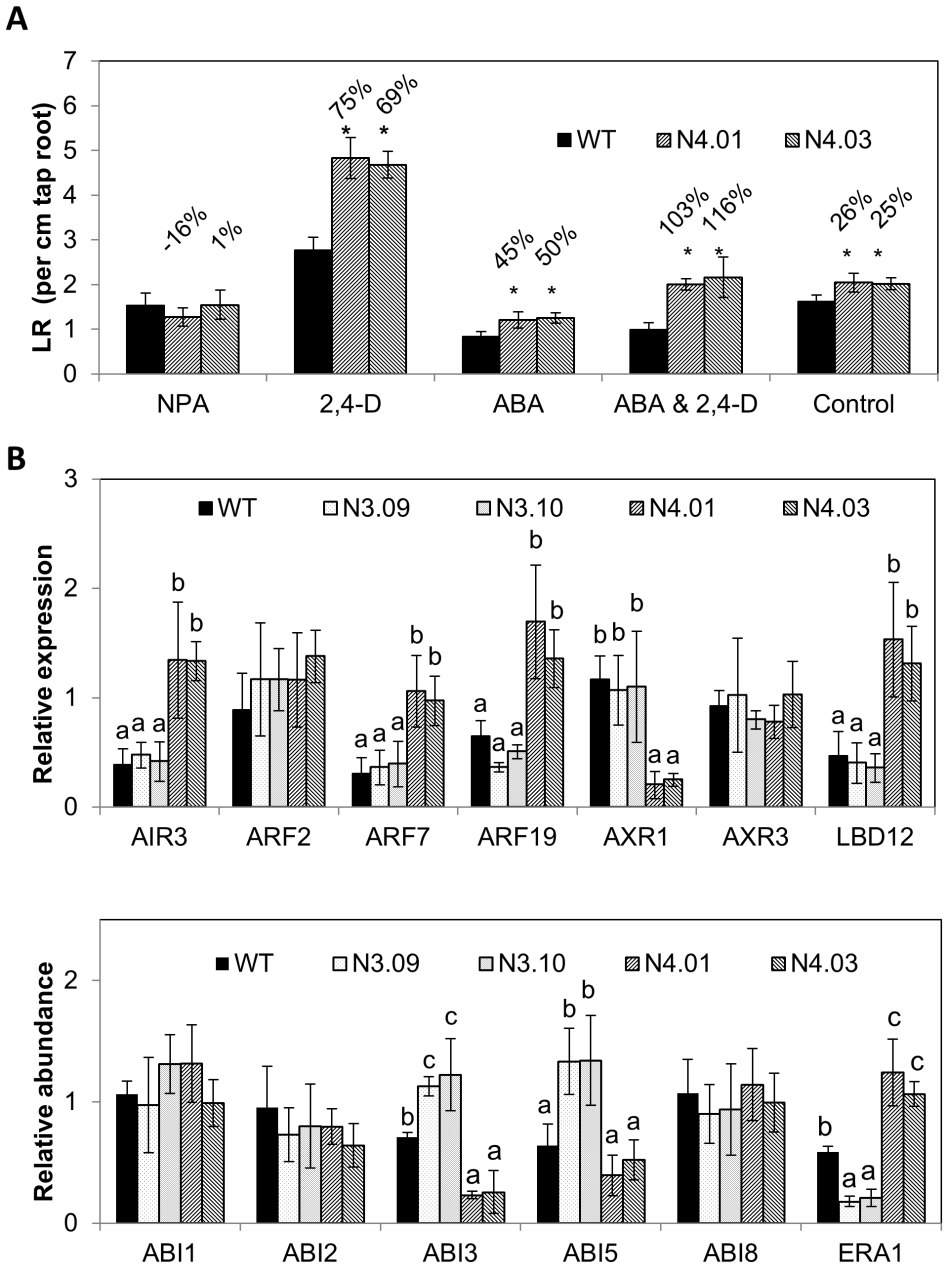
Role of GmNAC004 in regulation of lateral root density of transgenic *Arabidopsis* plants. (A) Transgenic *Arabidopsis* overexpressing *GmNAC004* in response to ABA and 2,4-D treatments. Four-day-old seedlings of WT and *GmNAC004* transgenic plants (T4 generation) were exposed to 5 µM ABA, 20 nM 2,4-D, 2 µM NPA or their combinations for 7 days. Control treatment was not treated with hormones. Error bars are the standard errors of the means from six replications. Asterisks denote significant differences at 95% between the WT and transgenic plants. (B) Expression of representative ABA and auxin signaling genes in transgenic GmNAC003 and GmNAC004 *Arabidopsis* plants. Duncan multiple-mean comparisons were used and different letters indicates differences of the means.

The role of *GmNAC004* in counteracting the inhibition of seed germination by ABA (Figure 6) raises the possibility that the GmNAC004 protein may also counteract ABA inhibition of LR development, which has been reported under both normal and stress conditions [26], [28], [61]. This hypothesis was tested by examining the effects of applied ABA on LR development in the *GmNAC004* transgenic plants under well-watered conditions. Figure 7A shows that ABA suppressed LR density of both transgenic and WT plants, but the suppression was lower for the transgenic plants. This result suggests that GmNAC004 partially represses ABA-induced inhibition of LR number.

The expression of *GmNAC004* was induced by 2,4-D (Figure 2); therefore, it is possible that the role of GmNAC004 in regulation of LR number also involves auxin. To study the interaction of GmNAC004 and auxin signaling on LR development, we applied the synthetic auxin 2,4-D and the polar auxin transport inhibitor NPA to the growing media and measured root growth traits. We observed that under the control treatment, LR density was higher in the *GmNAC004* transgenic plants compared to the WT (Figure 7A). In the NPA treatment, LR density was significantly reduced in both the transgenic and WT plants. The NPA treatment totally suppressed the action of GmNAC004 in LR promotion so that there was no difference in LR density between the transgenic and WT plants. Furthermore, in the treatments with 2,4-D, either alone or together with ABA, overexpression of *GmNAC004* resulted in greater promotion of LR density in the transgenic plants in comparison with the WT. Taken together, these results demonstrate that the stimulation of LR development by GmNAC004 requires auxin and that GmNAC004 stimulates LR development via ABA-dependent and auxin-dependent signaling pathways.

To further elucidate the involvement of GmNAC004 in regulation of LR development, expression of a number of representative genes in ABA and auxin signaling pathways were quantified. The genes regulating auxin signaling include *AIR3*, *ARF2*, *ARF7*, *ARF19, AXR1*, *AXR3*, and *LBD12*, and ABA signaling include *ABI1*, *ABI2*, *ABI3*, *ABI5*, *ABI8* and *ERA1*. Figure 7B shows that *Arabidopsis* transgenic GmNAC004 plants had elevated expression of a number of auxin signaling genes including *AIR3*, *ARF7*, *ARF19*, and *LBD12*, while reduced expression of *AXR1*. Expression of a number of genes in ABA signaling was also altered, with increased expression of a negative regulator of ABA response gene *ERA1*. GmNAC003 overexpression, in contrast, suppressed expression of *ERA1* while stimulation of *ABI3* and *ABI5*, indicating a positive regulator of ABA signaling. Alteration of auxin and ABA related genes indicates that GmNAC004 may regulate LR development through auxin and ABA signaling.

## Discussion

Significant advances in understanding of molecular networks regulating LR development in the past few years enabled us to further investigate the molecular regulation of root architecture under water deficit. The promotion of numbers and length of LRs in lower soil profiles in response to water deficit, which has been reported previously [2]–[4], [13], is considered an avoidance mechanism of soybean plants to water stress conditions [9]. This occurrence must be regulated by the activation of responsive proteins that control growth and optimize development to adapt to the stress. Using transcriptional profile analysis, we characterized two soybean genes, *GmNAC003* and *GmNAC004*, which were expressed strongly in roots and leaves of soybean in response to water stress. Strong lines of evidence have indicated that NAC TFs play an important role in water stress responses and in plant development [29], [62]–[65]. In *Arabidopsis*, individual overexpression of the dehydration-inducible genes *ANAC019*, *ANAC055* and *ANAC072* improved water stress tolerance [27]. In rice, Hu *et al.* [34] reported that transgenic plants overexpressing a drought-inducible *SNAC1* gene could maintain leaf turgor and better spikelet fertility under drought stress. In regulation of LR development, NAC TFs are promoters of LR numbers in *Arabidopsis* and soybean. *Arabidopsis* NAC1 and AtNAC2 transcription factors stimulate LR development through auxin signaling [38], [39]. In addition, *AtNAC2* was up-regulated by ABA and salinity treatments, suggesting the interaction of root development and osmotic stress. In soybean, Hao *et al.* [37] recently reported that a stress inducible GmNAC20 stimulated LR development and stress tolerance in the *Arabidopsis* heterologous system. Molecular analysis of transgenic plants overexpressing *GmNAC20* revealed that the gene regulated several genes in the auxin signaling network including auxin response factor genes *ARF7* and *ARF19*, and a lateral boundaries domain gene *LBD12*. GmNAC20 TF also induced a number of cold-responsive genes. In the present study, we demonstrated that *GmNAC004* was induced by water stress, ABA and auxin. These results suggest that GmNAC004 transcription factor have a role in regulation of plant development in response to environmental stress.

Using *Arabidopsis in planta* study, a role of GmNAC004 in governing LR number and development was apparent. Figures 4B & 5 show that GmNAC004 significantly promoted LR number under non-stress conditions and that the transgenic plants were able to maintain higher LR numbers and total LR length than the WT under mild water deficit conditions. In contrast, although having a relative conserved DNA binding domain and transcription activation activity [47] when compared with GmNAC004, GmNAC003 did not promote LR number. The difference in phenotypes of GmNAC003 and GmNAC004 transgenic plants might be due to the highly divergence of the sequences of the transcription activation domains at the C-terminal of the proteins.

The involvement of GmNAC004 in regulation of LR development through the ABA- and auxin-signaling pathways was also evident. The suppression of ABA inhibition of seed germination by GmNAC004 (Figure 6) indicates that GmNAC004 counteracts ABA signaling. Experiments using ABA treatments of transgenic GmNAC004 and WT *Arabidopsis* further demonstrated that the protein is involved in suppression of ABA inhibition of LR number. ABA treatments (ABA, ABA & 2,4-D) dramatically inhibited LR numbers in both the transgenic and WT lines, but the inhibition was not as strong as in the transgenic plants overexpressing *GmNAC004* (Figure 7A). The role of GmNAC004 in regulation of LR development is supported by the report of De Smet *et al.* [26], wherein they proposed a model of ABA-auxin interaction in the regulation of LR development: ABA inhibits LR development while auxin promotes it. This function of GmNAC004 might work at least under normal growing conditions in which the inhibitory effect of ABA on LR number has been reported. Deak and Malamy [28] found that the ABA-deficient mutants of *Arabidopsis aba2-1* and *aba3-1* showed higher numbers of LRs than the WT under both well-watered and water-stressed (artificially induced by mannitol treatment) conditions. A similar observation was reported in an *Arabidopsis* ABA-deficient mutant in which the ABA biosynthesis enzyme 9-*cis*-epoxycarotenoid dioxygenase (NCED) was impaired. The *nced3* mutant showed an increased LR number under well-watered conditions [61]. In contrast to ABA, auxin is a positive regulator of all stages of LR development from pre-initiation to emergence [17]. In our experiments, *GmNAC004* was up-regulated by 2,4-D treatment in soybean seedlings (Figure 2), and the transgenic *Arabidopsis* plants overexpressing *GmNAC004* showed a greater promotion of LR density when compared with the WT treated with 2,4-D (Figure 7A). In addition, *GmNAC004* transgenic plants showed no promotion of LR number when auxin transport was blocked by NPA treatment, indicating that an auxin gradient is required for the action of GmNAC004 in the regulation of LR number. In supporting for this response, molecular analysis showed that GmNAC004 appears to regulate downstream molecules in both ABA and auxin signaling pathways. GmNAC004 promoted expression of *AIR3*, *ARF7*, *ARF19* and *LBD12* and reduced expression of AXR1 (Figure 7B). *AIR3* was an auxin responsive gene which acts downstream of *Arabidopsis* NAC1 and soybean GmNAC20 in promotion of LR development in *Arabidopsis* [37], [39]. ARF7 and ARF19 belong to the ARF family of transcriptional factors which bind to auxin response elements in the promoters of auxin-responsive genes. Double mutant arf7/arf19 severely reduced LR formation in *Arabidopsis* and significantly impaired global expression of auxin responsive genes [66]. LBD12 is involved in the formation of lateral organs and differentially expressed in roots [67]. AXR1 encodes an E1 ubiquitin-activating enzyme targeting degradation of the AUX/IAA transcriptional repressors in response to auxin and might have a role in regulating LR in *Arabidopsis* [68]. In contrast to the positive role in regulation of auxin signaling pathway, GmNAC004 down-regulates expression of *ABI8* and *ERA1* in the ABA signaling pathway. ABI8 is differentially expressed in root elongation zone of *Arabidopsis* and has a function in ABA-regulated seed dormancy and maintenance of root meristem [69], [70]. ERA1 is a farnesyl transferase which acts as a negative regulator of ABA response in regulation of seed germination and stomata opening, and the knockout mutant *era1* resulted in increase in lateral root number [71]. In contrast, GmNAC003 suppressed expression of *ERA1* while promoted *ABI3* and *ABI5* to enhance ABA signaling [71]. Collectively, these data indicate that GmNAC004 may enhance auxin signaling but suppress ABA signaling to regulate LR development in *Arabidopsis*. The results show an additional crosstalk between the auxin and ABA signaling pathways and are in agreement with the views of De Smet *et al.* [26] on the antagonistic roles of auxin and ABA in the regulation of LR development.

Although transgenic *Arabidopsis* plants overexpressing *GmNAC004* had greater LR number and length than the WT under both well-watered and mild water stress conditions, they did not show promotion of LR in response to water stress when compared to the well-watered condition (Figure 4B). Thus, the results of this study do not support a role of GmNAC004 in the enhanced LR number under water stress in soybean that has been reported previously [2], [4]. There are two possibilities: (i) the soybean GmNAC004 protein does not have a function in increased LR development in soybean under water stress; or (ii) it has a function, but it is unable to increase LR number in *Arabidopsis* under water stress. The first possibility is supported by a similar study in *Arabidopsis* under salt stress [38]. The authors found that NaCl stress increased LR numbers of *Arabidopsis*. Under both normal and mild salinity stress conditions, transgenic plants overexpressing *AtNAC2*, a salt stress-responsive gene, had greater LR numbers than the WT. However, LR number of *AtNAC2* transgenic *Arabidopsis* plants was not enhanced in the NaCl stress compared to the non-stress condition. In contrast, the *nac2* mutant showed a similar response to the WT. This suggests that AtNAC2, although a promoter of LR number under normal conditions and induced by saline stress, was not the protein that regulated the enhanced LR number in *Arabidopsis* in response to salt stress. The second possibility is that *Arabidopsis* lacks of the regulatory system which supports a gene, either native or heterologous as GmNAC004 to act on promotion of LR number under water deficit stress. This is supported by the fact that LR development in *Arabidopsis* is significantly inhibited in response to low water potential conditions induced by mannitol [28] or by PEG [59]. In contrast to *Arabidopsis*, soybean shows stimulation of LR development under water deficit conditions [2], [4]. Therefore, in soybean, there may be a regulatory system that supports gene action to promote LR number under mild water deficit. To investigate this possibility, it would be useful to study the function of GmNAC004 in the enhancement of LR number using transgenic soybean.

In conclusion, the findings of this study indicate that the soybean GmNAC004 TF has a role in stimulating LR number and total LR length under non-stress conditions and maintains greater LR development under mild water stress, compared to the WT. However, LR development of GmNAC004 transgenic *Arabidopsis* plants was not enhanced in the water-stressed compared to the well-watered treatment. Nevertheless, *GmNAC004* can potentially be used for genetic engineering for a higher lateral root number, at least under normal growth conditions. Plants with early development of a large and extensive root system may benefit during later growth stages, where water deficit may occur. These plants can tolerate to drought by the dehydration avoidance mechanism to maintain tissue water content [9], [72] that helps the plant survive and reproduce under drought conditions.

## Supporting Information

Figure S1 **Phylogenetic tree of soybean NAC proteins according to Le et al. (2011).** Tree was drawn using Mega 5.1 software (Tamura et al. 2011) with a bootstrap of 500 replicates. GmNAC20 (Hao et al. 2011) and GmNAC003 and GmNAC004 (this paper) were noted on right side, adjacent to the names assigned by Le et al. (2011).
(PDF)

Table S1 **Soybean W82 water stress treatments.** For the water stress treatments, the plants were not supplied with water after they reached the V1 growth stage (14 days after sowing). Water-stressed tissues were collected when the stem water potentials (Ψ_W)_ reached −0.5, −1.0, and −1.5 MPa. The stressed plants did not produce more leaves while the corresponding controls reached the V2, V3 and V6 growth stages, respectively.
(DOCX)

Table S2 **Primers of reference genes,** ***GmNAC003*** **and** ***GmNAC004*** **used for qRT-PCR analysis in soybean and** ***Arabidopsis*** **.**
(DOCX)
