# Supplementary material for: Functional Analysis of Water Stress-Responsive Soybean GmNAC003 and GmNAC004 Transcription Factors in Lateral Root Development in Arabidopsis
Source: PLoS One. 2014 Jan 23;9(1):e84886. doi: 10.1371/journal.pone.0084886 (PMC3900428; doi:10.1371/journal.pone.0084886)
Supplement: Figure S1 — Phylogenetic tree of soybean NAC proteins according to Le et al. (2011). Tree was drawn using Mega 5.1 software (Tamura et al. 2011) with a bootstrap of 500 replicates. GmNAC20 (Hao et al. 2011) and GmNAC003 and GmNAC004 (this paper) were noted on right side, adjacent to the names assigned by Le et al. (2011). (PDF) [file pone.0084886.s001.pdf]

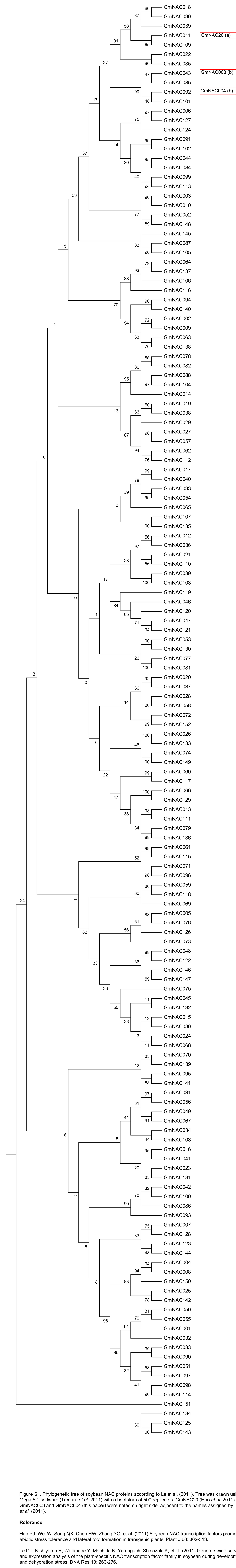

Figure S1. Phylogenetic tree of soybean NAC proteins according to Le et al. (2011). Tree was drawn using Mega 5.1 software (Tamura *et al.* 2011) with a bootstrap of 500 replicates. GmNAC20 (Hao *et al*. 2011) and GmNAC003 and GmNAC004 (this paper) were noted on right side, adjacent to the names assigned by Le *et al*. (2011).

Reference

Hao YJ, Wei W, Song QX, Chen HW, Zhang YQ, et al. (2011) Soybean NAC transcription factors promote abiotic stress tolerance and lateral root formation in transgenic plants. Plant J 68: 302-313.

Le DT, Nishiyama R, Watanabe Y, Mochida K, Yamaguchi-Shinozaki K, et al. (2011) Genome-wide survey and expression analysis of the plant-specific NAC transcription factor family in soybean during development and dehydration stress. DNA Res 18: 263-276.

Tamura K, Peterson D, Peterson N, Stecher G, Nei M, et al. (2011) MEGA5: molecular evolutionary genetics analysis using maximum likelihood, evolutionary distance, and maximum parsimony methods. Mol Biol Evol 28: 2731-2739.
