# Supplementary material for: Functional Analysis of Water Stress-Responsive Soybean GmNAC003 and GmNAC004 Transcription Factors in Lateral Root Development in Arabidopsis
Source: PLoS One. 2014 Jan 23;9(1):e84886. doi: 10.1371/journal.pone.0084886 (PMC3900428; doi:10.1371/journal.pone.0084886)
Supplement: Table S1 — Soybean W82 water stress treatments. For the water stress treatments, the plants were not supplied with water after they reached the V1 growth stage (14 days after sowing). Water-stressed tissues were collected when the stem water potentials (ΨW) reached −0.5, −1.0, and −1.5 MPa. The stressed plants did not produce more leaves while the corresponding controls reached the V2, V3 and V6 growth stages, respectively. (DOCX) [file pone.0084886.s002.docx]

**Supplementary Table S1. Soybean W82 water stress treatments**. For the water stress treatments, the plants were not supplied with water after they reached the V1 growth stage (14 days after sowing). Water-stressed tissues were collected when the stem water potentials (Ψ_W)_ reached -0.5, -1.0, and -1.5 MPa. The stressed plants did not produce more leaves while the corresponding controls reached the V2, V3 and V6 growth stages, respectively.

| Days from sowing | Control plants | | Water stressed plants | | |
| --- | --- | --- | --- | --- | --- |
|  | Growth stage | Stem Ψ_W_ (MPa*) | Growth stage | Stem Ψ_W_ (MPa*) |  |
| 18 | V2 | -0.25 | V2 | -0.50 | |
| 22 | V3 | -0.25 | V2 | -1.00 | |
| 34 | V6 | -0.25 | V2 | -1.50 | |

*Data ranged within ~ ±0.1 MPa
