# Supplementary material for: Functional Analysis of Water Stress-Responsive Soybean GmNAC003 and GmNAC004 Transcription Factors in Lateral Root Development in Arabidopsis
Source: PLoS One. 2014 Jan 23;9(1):e84886. doi: 10.1371/journal.pone.0084886 (PMC3900428; doi:10.1371/journal.pone.0084886)
Supplement: Table S2 — Primers of reference genes, GmNAC003 and GmNAC004 used for qRT-PCR analysis in soybean and Arabidopsis . (DOCX) [file pone.0084886.s003.docx]

**Supplementary Table S2. Primers of reference genes, *GmNAC003* and *GmNAC004* used for qRT-PCR analysis in soybean and *Arabidopsis*.**

| **Gene symbol** | **Annotation** | **NCBI Accession** | **Species** | **Primers** | **Amp. Length (bp)** |
| --- | --- | --- | --- | --- | --- |
| *ACT11* | Cytoskeletal structural protein | BW652479 | *G. max* | ATCTTGACTGAGCGTGGTTATTCC | 126 |
|  |  |  |  | GCTGGTCCTGGCTGTCTCC |  |
| *TIP41* | Target of rapamycin signaling element | EV263725 | *G. max* | AGGATGAACTCGCTGATAATGG | 88 |
|  |  |  |  | CAGAAACGCAACAGAAGAAACC |  |
| *TUB4* | Structural constituent of cytoskeleton | EV263740 | *G. max* | GGCGTCCACATTCATTGGA | 111 |
|  |  |  |  | CCGGTGTACCAATGCAAGAA |  |
| *UKN1* | Unknown | BU578186 | *G. max* | TGGTGCTGCCGCTATTTACTG | 74 |
|  |  |  |  | GGTGGAAGGAACTGCTAACAATC |  |
| *UKN2* | Unknown | BE330043 | *G. max* | GCCTCTGGATACCTGCTCAAG | 79 |
|  |  |  |  | ACCTCCTCCTCAAACTCCTCTG |  |
| *CYP2* | Cyclophilin | TC224926 | *G. max* | CGGGACCAGTGTGCTTCTTCA | 154 |
|  |  |  |  | CCCCTCCACTACAAAGGCTCG |  |
| *IDE* | Insulin degrading enzyme | AW310136 | *G. max* | ATGAATGACGGTTCCCATGTA | 114 |
|  |  |  |  | GGCATTAAGGCAGCTCACTCT |  |
| *SUBI-2* | Ubiquitin | D26092 | *G. max* | AGCTATTCGCAGTTCCCAAAT | 84 |
|  |  |  |  | CAGAGACGAACCTTGAGGAGA |  |
| *CDPK* | CDPK-related protein kinase | AW396185 | *G. max* | TAAAGAGCACCATGCCTATCC | 97 |
|  |  |  |  | TGGTTATGTGAGCAGATGCAA |  |
| *GmNAC003* | NAC transcription factor gene | DQ028771 | *G. max* | CCCTGCCACGAGTGAAC | 66 |
|  |  |  |  | TTCGGAGACCCGAATTTCT |  |
| *GmNAC004* | NAC transcription factor gene | DQ028772 | *G. max* | CGTCAGTTCCGCAAAAGAT | 62 |
|  |  |  |  | GACCCGTTGGTTTCTCAC |  |
| *UBQ5* | Ubiquitin extension protein | At3g62250 | *A. thialana* | AGAAGAAGACTTACACCAAGCCGA | 113 |
|  |  |  |  | CCTCAAACGCTGAACCTTTCC |  |
| *AIR3* | Auxin-induced in root cultures 3 | NM_126453.4 | *A. thialana* | GTTGTGTCTGTCTTCCCAAACA | 129 |
|  |  |  |  | ATCTTCTCCGAACCTAGCCTTT |  |
| *ARF2* | Auxin response factor 2 | AY669787.1 | *A. thialana* | GACGGTTCCTAGACAAGACGAC | 129 |
|  |  |  |  | CAGAGAAGCTTTGACGGAAGAT |  |
| *ARF7* | Auxin response factor 7 | AY669789.1 | *A. thialana* | TTTGCAACCACAATTAGTTTCG | 128 |
|  |  |  |  | AATGGTGATGTGCTTGTTGAAG |  |
| *ARF19* | Auxin response factor 19 | AY669794.1 | *A. thialana* | TTCGATGTTCCCTGGTTTAAGT | 130 |
|  |  |  |  | GGAAGCAAAATTGTTTGGTAGG |  |
| *AXR1* | Auxin resistant 1 | L13922.1 | *A. thialana* | AGGAGAATCCAGACACGTTGAT | 131 |
|  |  |  |  | CTTAACGTTTGCATCTCGACAG |  |
| *AXR3* | Auxin resistant 3 | AF040632.1 | *A. thialana* | TTCTCAGAGACGGTTGATCTGA | 130 |
|  |  |  |  | GTTTGGCTGGATCTTTAGGACA |  |
| *LBD12* | Lateral organ boundaries | NM_128568.1 | *A. thialana* | ATTCGCCATTGTTCATAAGGTC | 130 |
|  |  |  |  | CGGATCTCTTACTCTTGCGTTT |  |
| *ABI1* | abscisic acid-insensitive 1 | AY142623 | *A. thialana* | TGAAGAAGCGTGTGAGATGG | 159 |
|  |  |  |  | CTGTATCGCCAGCTTTGACA |  |
| *ABI2* | abscisic acid-insensitive 2 | AY136415.1 | *A. thialana* | CAGTCGCTGTTCCATTCAGA | 104 |
|  |  |  |  | CGCCAGAACAAGAACTTTCC |  |
| *ABI3* | abscisic acid-insensitive 3 | NM_113376.3 | *A. thialana* | GGGAGGGACCTGGATGTATT | 92 |
|  |  |  |  | GCTCGGTCCATGGTAGGTAA |  |
| *ABI5* | abscisic acid-insensitive 5 | NM_129185 | *A. thialana* | ACTTCCAGCTCCGCTTTGTA | 139 |
|  |  |  |  | GGTTGTCTAGCCGCAGTCTC |  |
| *ABI8* | abscisic acid-insensitive 8 | NM_111689 | *A. thialana* | CCTCTGCTTCTGTTCGTTCC | 131 |
|  |  |  |  | GATTTGAGCAGTCGGAGGAG |  |
| *ERA1* | Farnesyltransferase beta subunit | NM_123392 | *A. thialana* | CTGTGGTTTGGCTGCTATGA | 165 |
|  |  |  |  | TCGATGTACAGCCCAATTCA |  |
